# Hints as to Care of Infants

**Published:** 1901-06

**Authors:** C. E. Boynton

**Affiliations:** Sandy, Utah


### HINTS AS TO CARE OF INFANTS.

SANDY, UTAH, May 16, 1901.

*Atlanta J.-R. of Medicine:*

1. Give a baby plenty of water four or five times per day.
2. Give a baby a rockless bed to itself.
3. A baby should be held only to be nursed.
4. Massage and exercise a baby each day.
5. Give a baby a chance to lay naked and kick daily.
6. Never give a baby diluted alcohol, liquors or coffee; it will acquire perverted appetites soon enough.
7. Teach a baby to hang by its hands and spring with its legs.
8. Don't advise a parent to give the baby a little soothing syrup, paregoric or castor oil; in short, do no off-hand prescribing at all.
9. If the mother is weak or anemic, correct any lesion and give her calcium hypophosphite 5 to 20 grains per day; thus via milk nourish baby.
10. If the baby is constipated give the mother sodium phosphate q. s.
11. Ventilate sufficiently and keep temperature below 75° F.
12. A cart load of sand makes a healthful plaything for a baby.

C. E. BOYNTON, M.D.

